# Assessment of malaria prevention knowledge, attitude, and practice and associated factors among households living in rural malaria-endemic areas in the Afar Pastoral Region of Ethiopia

**DOI:** 10.3389/fpubh.2023.1258594

**Published:** 2023-10-20

**Authors:** Desalegne Addis, Temesgen Gebeyehu Wondmeneh

**Affiliations:** Department of Public Health, College of Medical and Health Science, Samara University, Afar, Ethiopia

**Keywords:** knowledge, attitude, practice, malaria, household, endemic, Afar, Ethiopia

## Abstract

**Background:**

Malaria morbidity has reduced significantly in most regions of Ethiopia, but it is still a serious issue in the northeast, particularly in the Afar region.

**Objective:**

The study aimed to evaluate household heads' knowledge, attitudes, and practices toward malaria prevention and its associated factors in rural Ada'ar woreda district in the Afar region.

**Methods:**

A community-based cross-sectional study was conducted among 422 households living in Ada'ar woreda district. A systematic sampling technique was used to select households. A pre-tested, structured questionnaire was used to interview randomly selected adult household heads. Frequency and percentage were computed. Logistic regression was used to determine the association between independent and dependent variables. Statistical significance was considered to be a *p*-value <0.05.

**Results:**

Nearly two-thirds (64.2%) of household heads had good knowledge of malaria prevention, and 46.9% had a positive attitude toward it. About 17.3 and 56.9% of study participants had good malaria prevention practices and good healthcare seeking behaviors, respectively. Illiterate (AOR = 2.62, 95% CI: 1.49–4.63) and low-income (AOR = 2.6, 95% CI: 1.2–5.6) participants were more likely to have poor knowledge of malaria prevention (malaria signs and symptoms, malaria transmissions, and malaria prevention methods). Married participants (AOR = 2.52, 95% CI: 1.02–6.29) and illiterates (AOR = 2.83, 95% CI: 1.69–4.73) had negative attitudes toward malaria prevention. Household heads with poor knowledge of malaria prevention had 85% higher rates of practicing poor malaria prevention methods (regular bed nets used; AOR = 1.85, 95% CI: 1.2–2.8). Young adults (18–25 years) were more likely to have poor healthcare seeking behaviors (AOR = 3.5, 95% CI: 1.73–7.1), while pastoralists had a lower likelihood (AOR = 0.46, 95% CI: 0.28–0.8).

**Conclusion:**

Knowledge, attitude, and practices toward malaria prevention remain a problem in malaria-endemic rural areas of the Afar region of Ethiopia. There is a need for the implementation of interventions that will focus on increasing knowledge of malaria prevention and encouraging positive attitudes toward it, as well as promoting regular bed net usage and healthcare seeking behaviors.

## Background

Malaria is a vector-borne disease endemic in most of sub-Saharan Africa, where the most common parasite to infect humans is *Plasmodium falciparum* (1). It is a disease with complicated patterns of transmission that is linked to significant geographical and temporal variation (2). Malaria poses a threat to millions of people living in tropical and subtropical countries (3). Throughout the world, malaria causes 300–500 million cases and up to three million fatalities annually; of these, Africa alone bears more than 90% of the burden, and more than 80% of malaria deaths take place there, while < 15% occur in Asia and Eastern Europe (4). Globally, malaria cases increased by 5.8% and deaths by 11% in 2020 compared to 2019. Two-thirds of the additional deaths in 2020 compared to 2019 were attributable to the disruption in malaria prevention and control efforts brought on by the COVID-19 pandemic (5). East Africa is an important region to focused on in the global fight against malaria because it accounted for 25% of all cases worldwide (6). In Djibouti, the prevalence of malaria was 70.8% (7). In Ethiopia, the pooled prevalence of malaria among adults was 13.6% (8). In the Afar region, the prevalence of malaria among febrile under-five children was 64% (9). Seasonal malaria transmission persists when the maximum monthly rainfall is < 600 mm and the temperature is markedly above 15°C or below 40°C (10). The use of medication and bed nets is an efficient malaria prevention strategy that can be implemented in malaria-endemic areas (11). The government of Ethiopia distributes the bed nets through an ongoing effort campaign every three years (12). Malaria infection depends on people's knowledge and awareness of the disease (13). Understanding the biology of malaria transmission at the individual level is an essential aspect of the strategies used to stop the spread of malaria parasites (14). In sub-Saharan Africa, schoolchildren's knowledge of malaria's causes and transmission ranged from 19.2 to 85%, and 51.2% of them had a positive attitude. Schoolchildren practice low to moderate levels of malaria prevention, ranging from 32.4 to 67.9% (15). In Senegal, nearly a third of adolescents had good knowledge of malaria (34.4%) and good practice for using bed nets (32.8%), whereas 59.0% had a positive attitude and 73.8% had good care-seeking practice toward malaria (16). Adults in Nigeria had a high level of comprehensive knowledge (90%) and practiced malaria prevention (80%). Seeking hospital care was a good practice (68.5%), while attitudes about antimalarial treatment were poor (56.7%) (17). In Cameroon, 88% of pregnant women and mothers had a good level of knowledge, 99% had a good attitude toward ITNs, and 57% of respondents used ITNs to prevent malaria (18). Women of reproductive age in Burkina Faso had 56.1% accurate knowledge of the preventive measures, causes, and symptoms of malaria. About 97.4% of women said they slept under a mosquito net, and most of the women (80%) reported sleeping within an insecticide-treated net (19). In South Africa, 63% of household heads were able to identify at least three symptoms of malaria. Participants' attitudes toward indoor residual spraying (IRS) were favorable for 76% of respondents. Only 2% of the participants used bed nets (20). In Mozambique, 90.0% of households' heads had knowledge of malaria prevention. About 81.7% of respondents slept under an ITN at night (21). In Tanzania, 47.3 and 13.8% of household heads had moderate and high-level knowledge of malaria prevention, respectively. The majority of the households (83.9%) had bed nets hanging on the sleeping spaces, while 95% of household heads agreed that it was beneficial to sleep beneath a bed net (22). In a study of Southern Ethiopia, household heads who had good knowledge, a positive attitude, and good practices toward malaria prevention were 50.4%, 55.1%, and 67.7%, respectively (23). Approximately two-thirds of participants in the Amara region of Ethiopia had good knowledge (63.1%) and a positive attitude (62.6%) toward malaria, whereas only half had good practice (50.8%) with regard to malaria prevention and control measures (24). In a study of the Oromia region in Ethiopia, about 85% of household heads had good knowledge of malaria prevention, 87.8% had a positive attitude toward malaria prevention, and 51.3% regularly practiced malaria prevention (25). Self-medication was practiced by some people to prevent malaria (26). The majority of Afar people (71%) preferred to receive their medical care from modern health facilities, 16% used traditional practices, and 3% used self-medication during their symptoms of malaria. The use of traditional medicine may increase as people get older, whereas the use of modern medication rose as people's educational levels increased (27). Education and knowledge are used to combat malaria (28). Poor knowledge resulted in poor management of malaria (29). A person's knowledge about malaria prevention was affected by male gender, rural residence, low income level, and illiterate educational level (16, 18, 19, 22–24). Male gender, rural residence, low educational level, low income (16, 24, 30), and Islamic religion (23) were associated with lower rates of use of malaria prevention measures, whereas good knowledge boosted the use of malaria prevention practices (31, 32). A person's attitude toward malaria prevention was affected by a low wealth quintile and a low educational level (16).

Even though malaria is endemic and has a seasonal outbreak in the pastoral region of Afar, there is no data on knowledge, attitudes, or practices about malaria prevention in the region. Therefore, the current study investigated malaria-related knowledge, attitudes toward the disease, adoption of prevention practices, and care-seeking practices among household heads living in rural areas with persistent malaria transmission in the Afar region. The study also explored factors associated with household heads knowledge, attitudes, and practices toward malaria.

The aim of the study was to generate data that will aid in the development of social and behavioral change interventions targeted specifically at household levels in order to improve households' awareness and adherence to malaria control and preventative measures in the Afar region.

## Methods

### Study area and design

A community-based cross-sectional study design was conducted in Ada'ar woreda, located in Zone 1 (also known as Awsi Rasu), Afar Region, northeast Ethiopia, from February 30th to May 10th, 2023, to assess knowledge, attitude, and practice of malaria prevention and control. There were five zones in the Afar region, with Zone 1 (Awsi Rasu Zone) being one of them. Geographically, the region is located between 9°N−12°N latitude and 40°E−42°E longitude at the northern tip of the Great East African Rift Valley and at 432 m altitude. It is a pastoral region in north-eastern Ethiopia, 576 km from Addis Ababa. The regional weather condition is sunny, and recurrent natural disasters like drought and flooding are common in the region. Poverty remains high and multidimensional in the region. The population's mean index of health care deprivation was 64.6%, which indicates that the majority of the population did not consult any medical practitioner within a year. The absence or scarcity of health centers and practitioners and the inability of households to access health services due to financial and other constraints were contributing factors (33). According to the Ada'ar woreda Health Bureau report, there were 13 kebeles in the woreda with a total estimated population of 69,111. The study was conducted in six rural kebeles out of the 13 kebeles (Table 1).

**Table 1:** The total population and total households for the selected kebeles.

**Selected kebeles**	**Total populations**	**Total households**
Ada'ar	7,054	841
Siylu na Woki	5,517	968
Burka	5,677	902
Ledi	4,331	618
Woranso na Hormati	6,283	1,102
Hado	4,355	764

**Figure 1:**
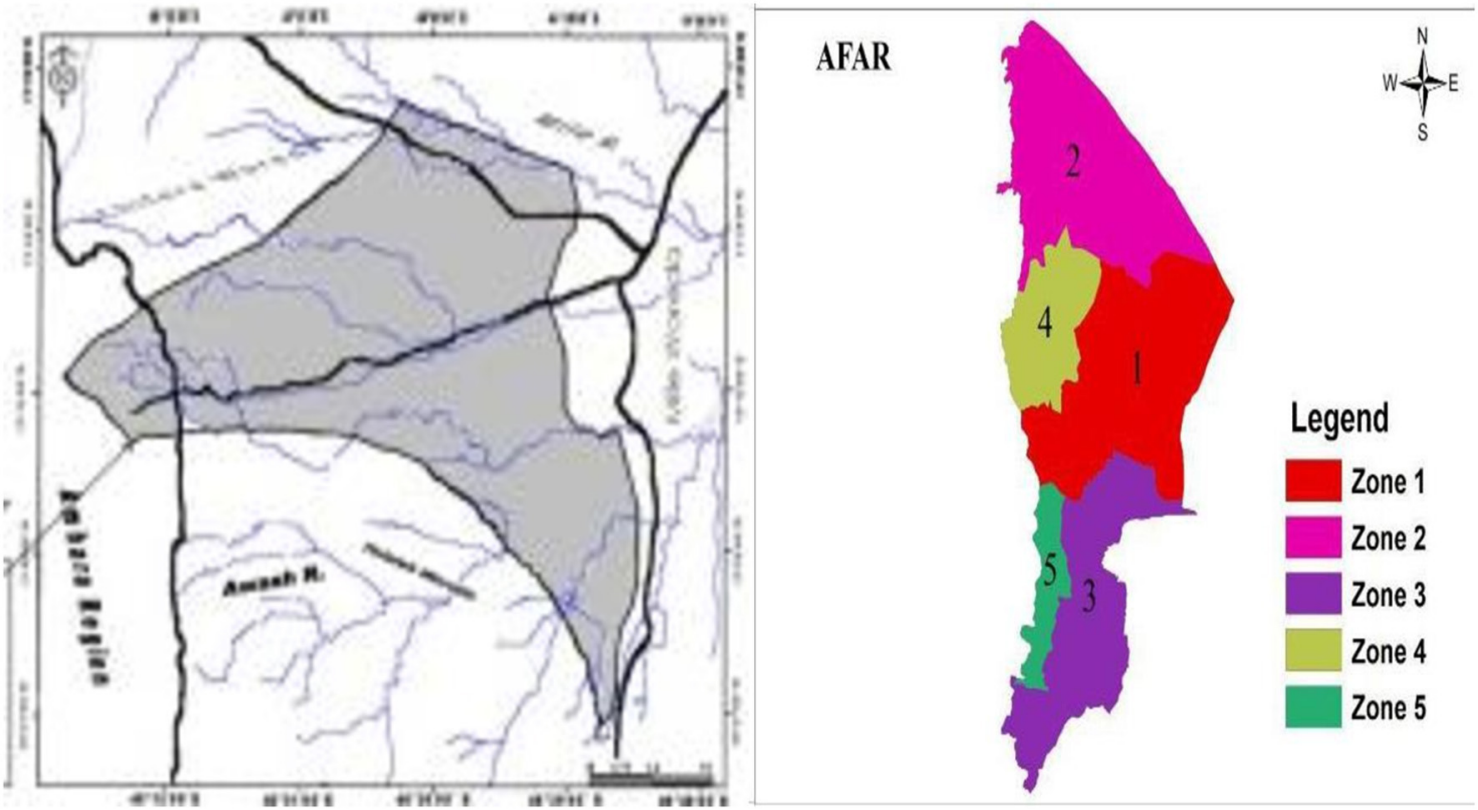
Map of the study zone.

There are three health centers, 12 health posts, and three private clinics in the woreda. The map of the study zone in the Afar region is depicted in Figure 1.

### Sample size determination

To determine the sample size of this study, we put the prevalence (*p*) at 50% because of the lack of existing research on the knowledge, attitudes, and practices of malaria prevention and control in the Afar region. With a confidence interval (CI) of 95%, precision (*d*) of 5%, and *q* of 50%, the sample size became 384. With the addition of a 10% non-response rate, the final sample size became 422.

### Source and study population

The source populations were all households in Ada'ar woreda, whereas the study populations were all households in the selected kebeles.

*Inclusion and exclusion criteria for study participants*: study participants should live permanently in the selected districts, and interviewees should be at least 18 years old (head of household), regardless of gender. Participants who are seriously ill or mentally ill, unable to communicate, or refuse to give consent were excluded from the study.

### Sampling method and procedure

For this study, Ada'ar woreda in Zone 1 (Awsi Rasu Zone) of the Afar region was selected purposefully because of the representativeness of the population dimension and the densely populated nature, which enabled the recruitment of an adequate sample. To ensure representativeness, the six kebeles were first selected by simple random sampling methods using lottery methods. Then the sample size is allocated proportionally to each of the six kebeles. The households in each kebele were selected using the following technique: initially, a list of households (a sampling frame) was developed by assigning a number to each household. Next, the sample interval (the number of households divided by the sample size) was calculated for each kebele, and a random start number was picked. Finally, from this first random number, households were systematically selected using the sampling interval until the calculated sample size was met. Interviews were conducted with the head of the selected household regardless of sex, and in the absence of the head of the household, a responsible person over 18 years old who had been appointed by the family was interviewed. When there are two or more heads in one household, the lottery method is used to select interview participants.

### Data collection methods and tools

The study questionnaires were adapted from various similar prior studies and reviewed. The questionnaire was first designed in English, and then translated into Afar, the local language in the study area. Closed questions on the following topics were included in the questionnaire: (i) socio-demographic characteristics of participants; (ii) knowledge of malaria transmission, symptoms, and signs of malaria and how to prevent malaria; (iii) practices for prevention; (iv) attitudes toward malaria; (v) practices for seeking care for malaria; and (vi) information channels. Five percent of the questionnaires were pre-tested among respondents in Chifra, a location not in the study area, to ensure word consistency, and some words were modified in light of the findings of the pilot study. Data was collected by prior-experienced data collectors. During the training of data collectors, the study protocol, the questionnaire, and the consent procedure were covered. Monitoring visits were made during the data collection phase to examine the accuracy of the data and determine whether the study's informed consent standards were being followed. In the event that the head of the household was not present, a responsible adult who is at least 18 years old was selected to take part in the interview.

### Operational definition and measurement of outcome variables

In this study, Kebele is the smallest administrative unit contained within a woreda in Ethiopia (34).

Malaria: Malaria diagnosis includes a patient's clinical assessment (for non-malaria endemic areas, fever, headache, chillness, fatigue, and vomiting; for malaria-endemic areas, only fever is suspected), microscopic examination of blood slides, and rapid diagnostic test (RDT) in accordance with the level of the health facility. Microscopic diagnosis remains the standard of diagnosis in health centers and hospitals of different levels, whereas multi-species rapid diagnostic tests (RDTs) are the main diagnostic tool at the health post level. Artemisinin-based combination therapies (ACTs) are the first-line drug for the treatment of uncomplicated *P. falciparum* malaria. Chloroquine is used for the treatment of *Plasmodium vivax* (35).

The primary outcomes investigated in this study were knowledge and attitudes about malaria, use of malaria prevention and control practices, and practices for seeking medical care when feeling ill. Participants' knowledge of malaria was assessed using three items: correctly identifying the mode of transmission, recognizing the sign and symptoms of malaria, and identifying methods to prevent malaria. The participant's scores on the three knowledge questions were added up to produce a knowledge score. Participants received a score of one if they mentioned a mosquito bite as the method of malaria transmission; otherwise, they received a score of zero. One point was given if at least three of the five basic symptoms of malaria—fever, headache, chills, fatigue and vomiting—were correctly identified. Participants who correctly identified a bed net as a method of preventing malaria were given a score of one, and those who correctly identified any of the other prevention methods—mosquito coils, wearing long clothes, applying insecticide spray, weeding, or disposing of sewage—were given a half-point. The overall score's median was used as the cutoff point for classifying knowledge of malaria into two levels. Participants' levels of malaria knowledge were rated as poor (below or equal to the median) or high (above the median). Six items pertaining to the population at risk for malaria, prevention, and treatment were used to measure attitudes about the disease. According to Likert's method of scoring, there were five possible responses: strongly agree (score 5), agree (score 4), neutral (score 3), disagree (score 2), and strongly disagree (score 1). For one item (malaria can be cured without medical care), the scale was reversed. An attitude score was estimated by adding up each participant's ‘score across the six variables. Participants were regarded as having a positive attitude if their total score was at or above the median; otherwise, they were considered to have a negative attitude. Participants who own a bed net and use it more than three times per week or every night are classified as practicing good prevention, while those who don't own a bed net or who do but don't use it more than three times per week are categorized as practicing poor prevention. We examined whether households actually required care within 24 h of the onset of symptoms in order to assess their behavior for seeking care for recent malaria. Households were deemed to have good care-seeking behavior if they visited a healthcare facility within 24 h of needing care; otherwise, they were deemed to have poor care-seeking behavior (16, 36, 37).

### Data management and analysis

Epidata version 3.1 was used to enter the data, which was subsequently exported to SPSS software version 26. For categorical variables, frequency and percentage were computed. Bivariate and multivariate logistic regressions were used to assess factors associated with household heads' knowledge, attitudes, and practices of malaria prevention. The multivariable regression models included variables that had a *p*-value of ≤0.25 in the bivariate analysis. Statistical significance was considered when the *p*-value <0.05.

## Results

### Socio-demographic characteristics of participants

The response rate for this study was 100%. Most of the respondents (39.8%) were in the age range of 26–35 years old. Male respondents were 55.9%. Married respondents constituted about 62.6%. The Muslim religion accounted for 82.2%. Most participants were pastoralists (74.6%). Illiterate respondents were 47.6%. About 25.8% of respondents had an average monthly income of 400–1,500 ETB (Table 2).

**Table 2:** Socio-demographic characteristics of participants.

**Variables**	**Categories**	**Frequency**	**Percentages (%)**
Age (in year)	18–25	85	20.1
	26–35	168	39.8
	36–45	102	24.2
	≥46	67	15.9
Sex	Males	236	55.9
	Females	186	44.1
Marital status	Married	264	62.6
	Single	134	31.8
	Divorced	24	5.7
Religious	Muslim	347	82.2
	Christian	75	17.8
Occupation	Pastoralist	315	74.6
	Agro pastoral	107	25.4
Educational level	Illiterate	201	47.6
	Primary school	115	27.3
	Secondary school	106	25.1
Average household income (ETB)	<2,000	58	13.7
	2,000–4,000	78	18.5
	4,001–6,000	109	25.8
	6,001–8,000	100	23.7
	>8,000	77	18.2

ETB, Ethiopian birr.

### Participants' information about malaria

**Figure 2:**
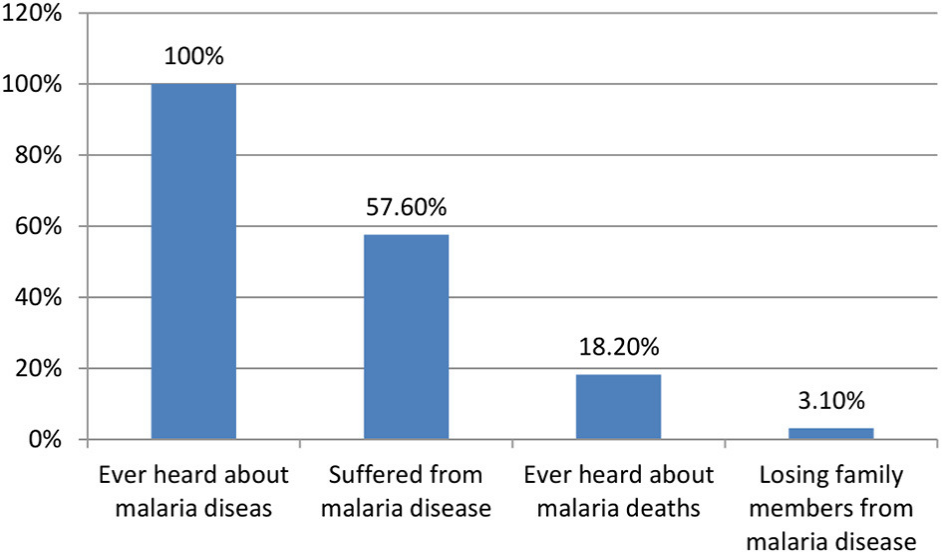
Participant's information regarding malaria.

All participants heard about malaria; 57.6% suffered from malaria disease, and 18.2% heard about malaria deaths. About 3.1% of participants lost family members from malaria (Figure 2).

### Source of information about malaria

**Figure 3:**
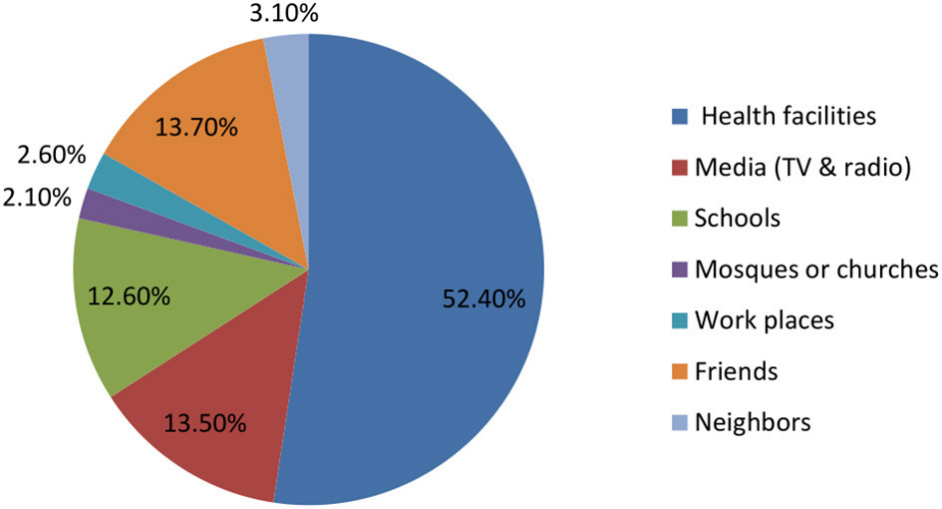
The source of information about malaria.

Health facilities were the source of malaria information for more than half (52.4%) of the study's participants. Nearly comparable percentages of study participants reported that the sources of information about malaria were the media (TV and radio; 13.5%), friends (13.7%), and schools (12.6%; Figure 3).

### Participants' knowledge of malaria transmission, signs and symptoms, and prevention methods

The majority of study participants (74.9%) reported that the transmission of malaria occurs through mosquito bites. Most respondents identified body pain (35.8%), fever (31.5%), and fatigue (27.5%) as malarial symptoms. Using mosquito bed nets was reported as a means of malaria prevention by the majority of respondents (43.1%). Nearly two-thirds (64.2%) of study participants had good knowledge of malaria (Table 3).

**Table 3:** Knowledge of malaria transmission, signs and symptoms, and prevention methods among participants.

**Variables**	**Categories**	**Frequency**	**Percentage (%)**
How is malaria transmitted?	Mosquito bite	316	74.9
	Contacting with malaria patients	61	14.5
	Using contaminated water	23	5.5
	I don't know	22	5.2
Malaria signs and symptoms	Fever	133	31.5
	Headache	97	23
	Vomiting	85	20.1
	Chilies	54	12.6
	Fatigue	116	27.5
	Body pain	151	35.8
	Feeling of thirsty	62	14.7
Malaria prevention	Using mosquito bed net	182	43.1
	Insecticide spray	94	22.3
	Using mosquito repellents lotion	31	7.3
	Using ventilators	66	15.6
	Avoiding stagnant water	49	11.6
Level of knowledge	Good	271	64.2
	Poor	151	35.8

### Attitude of participants toward malaria prevention

Nearly half of participants (52.4%, *n* = 221) believed that everybody can have malaria, and almost identical numbers also believed that malaria is deadly (53.3%, *n* = 225). Similarly, the same number of participants perceived that it is important to be tested before taking malaria treatment (49.3%, *n* = 208) and that it is necessary to finish malaria treatment (49.8%, *n* = 210). Half of the respondents (50.5%, *n* = 213) also stated that malaria can be prevented. Regarding the overall score of attitude, 46.9% of respondents exhibited a positive attitude (Table 4).

**Table 4:** Attitude of participants toward malaria prevention.

**Statements**	**Likert scales**				
	**Strongly disagree**	**Disagree**	**Neutral**	**Agree**	**Strongly agree**
	**No (%)**	**No (%)**	**No (%)**	**No (%)**	**No (%)**
Everybody can have malaria	49 (11.6)	74 (17.5)	78 (18.5)	118 (28)	103 (24.4)
Malaria can be cured without medical treatment	86 (20.4)	94 (22.3)	61 (14.5)	95 (22.5)	86 (20.4)
It is important to be tested before taking malaria treatment	70 (16.6)	64 (15.2)	80 (19)	113 (26.8)	95 (22.5)
Malaria can be prevented	77 (18.2)	74 (17.5)	58 (13.7)	127 (30.1)	86 (20.4)
It is necessary to finish malaria treatment	76 (18)	88 (20.9)	48 (11.4)	95 (22.5)	115 (27.3)
Malaria is deadly	66 (15.6)	84 (19.9)	47 (11.1)	80 (19)	145 (34.4)
Attitude score	Positive		198 (46.9%)		
	Negative		224 (53.1%)		

### Practices of participants for malaria prevention

About 41.9% of participants reported owning a bed net; of those, 7.3% reported using it every night, and 10% reported using it more than three days a week. Nearly one-fifth (21.1%) and 6.6% of study participants used insecticide spray and repellent lotion, respectively. About 13.5% of study participants considered ventilators for malaria prevention, and 16.8% never used any malaria prevention methods. Overall, 17.3% of participants had good malaria prevention practices (Table 5).

**Table 5:** Practices of participants for malaria prevention.

**Variables**	**Categories**	**Frequency**	**Percentages (%)**
Participants owned bed nets		177	41.9
Respondents' use of mosquito bed nets	Every night	31	7.3
	Over three days a week	42	10
	Three or less days per week	104	24.6
Insecticide spray		89	21.1
Using mosquito repellent lotion		28	6.6
Using ventilators		57	13.5
Never use any things		71	16.8
Family members slept under a bed net	All family members	73	17.3
	Fathers and mothers	31	7.3
	Children and mothers	43	10.2
	Mothers	15	3.6
	Children	15	3.6
Overall level of practices for malaria prevention	Good level of practice	73	17.3
	Poor level of practice	349	82.7

### Healthcare seeking behavior

**Figure 4:**
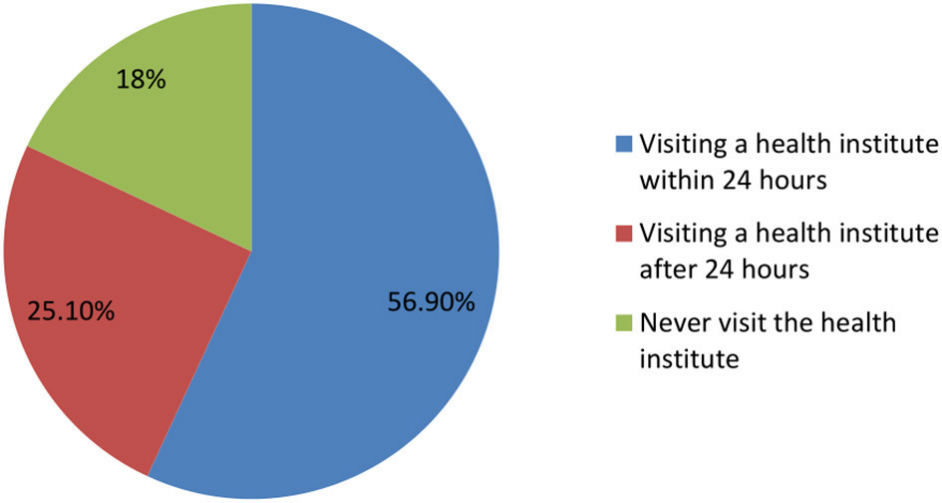
Care seeking practices of the study participants.

More than half of respondents (56.9%) reported visiting health facilities within 24 h after having malaria symptoms, while a quarter of respondents (25.1%) reported visiting health facilities more than 24 h after experiencing malaria symptoms. Eighteen percent of study participants never visited health facilities when they encountered malaria symptoms. Those study participants who attended a health facility within 24 h (56.9%) after experiencing malaria symptoms were regarded as having good care-seeking behavior for preventing malaria (Figure 4).

### Factors associated with malaria prevention knowledge

In the binary logistic regression analysis, the age range of 18–25 years, illiteracy, and an average household income of <2,000 ETB were significantly associated with poor knowledge of malaria prevention. In the multivariable analysis, participants with illiteracy (AOR = 2.62, 95% CI: 1.49 – 4.63) and low average monthly income (<2,000 ETB; AOR = 2.6, 95% CI: 1.2–5.6) were more likely to have poor knowledge of malaria prevention than those with secondary school and average monthly income >8,000 ETB, respectively (Table 6).

**Table 6:** Factors associated with malaria prevention knowledge.

**Variables**	**Categories**	**Knowledge**		**COR**	**AOR**
		**Good**	**Poor**		
		**No (%)**	**No (%)**		
Age (in year)	18–25	48 (11.4)	37 (8.8)	2.27 (1.13–4.56)^*^	1.92 (0.91– 4.0)
	26–35	103 (24.4)	65 (15.4)	1.86 (0.99–3.5)	1.82 (0.94–3.54)
	36–45	70 (16.6)	32 (7.6)	1.35 (0.67–2.68)	1.3 (0.64–2.71)
	≥46	50 (11.8)	17 (4)	1	1
Sex	Males	160 (37.9)	76 (18)	1.42 (0.95–2.1)	0.74 (0.48–1.13)
	Females	111 (26.3)	75 (17.8)	1	1
Educational status	Illiterates	108 (25.6)	93 (22)	3.11 (1.8–5.3)^*^	2.62 (1.49–4.63)^*^
	Primary	80 (19)	35 (8.3)	1.58 (0.86–2.9)	1.4 (0.8–2.61)
	Secondary	83 (19.7)	23 (5.5)	1	1
Average household monthly income (ETB)	< 2,000	22 (5.2)	36 (8.5)	3.6 (1.77–7.4)^*^	2.6 (1.2–5.6)^*^
	2,000–4,000	61 (14.5)	17 (4)	0.62 (0.3–1.27)	0.53 (0.25–1.1)
	4,001–6,000	69 (16.4)	40 (9.5)	1.28 (0.69–2.4)	1.3 (0.67–2.5)
	6,001–8,000	66 (15.6)	34 (8.1)	1.14 (0.6–2.15)	1.26 (0.65–2.46)
	>8,000	53 (12.6)	24 (5.7)	1	1

ETB, Ethiopian birr.

* Statistical significant.

### Factors associated with attitudes toward malaria prevention

In the binary logistic regression, illiterates and low household average monthly income were significantly associated with a higher negative attitude toward malaria prevention. In the multivariable logistic regression analysis, married participants (AOR = 2.52, 95% CI: 1.02–6.29) and illiterate participants (AOR = 2.83, 95% CI: 1.69–4.73) were more likely to have negative attitudes toward malaria prevention than study participants who were divorced and secondary school, respectively (Table 7).

**Table 7:** Factors associated with attitudes toward malaria prevention.

**Variables**	**Categories**	**Attitude**		**COR**	**AOR**
		**Positive**	**Negative**		
		**No (%)**	**No (%)**		
Age (in years)	18–25	34 (8.1)	51 (12.1)	1.64 (0.86–3.1)	1.27 (0.64–2.55)
	26–35	71 (16.8)	97 (23)	1.45 (0.846–2.64)	1.44 (0.79–2.62)
	36–45	58 (13.7)	44 (10.4)	0.83 (0.45–1.54)	0.766 (0.4–1.47)
	≥ 46	35 (8.3)	32 (7.6)	1	1
Marital status	Married	118 (28)	146 (34.6)	2.1 (0.87–4.9)	2.52 (1.02–6.29)^*^
	Single	65 (15.4)	69 (16.4)	1.77 (0.72–4.32)	1.9 (0.75–4.96)
	Divorced	15 (3.6)	9 (2.1)	1	1
Household occupation	Pastoralist	153 (36.3)	162 (38.4)	0.77 (0.49–1.2)	0.91 (0.56–1.47)
	Agro pastoralist	45 (10.7)	62 (14.7)	1	1
Educational status	Illiterates	71 (16.8)	130 (30.8)	3.02 (1.86–4.9)^*^	2.83 (1.69–4.73)^*^
	Primary	61 (14.5)	54 (12.8)	1.46 (0.85–2.5)	1.47 (0.84–2.55)
	Secondary	66 (15.6)	40 (9.5)	1	1
Household average monthly income (ETB)	< 2,000	18 (4.3)	40 (9.5)	2.5 (1.24–5.2)^*^	1.85 (0.86–3.99)
	2,000–4,000	35 (8.3)	43 (10.2)	1.4 (0.74–2.63)	1.31 (0.66–2.59)
	4,001–6,000	53 (12.6)	56 (13.3)	1.2 (0.67–2.2)	1.1 (0.6–2.1)
	6,001–8,000	51 (12.1)	49 (11.6)	1.09 (0.6–1.98)	1.2 (0–62–2.17)
	>8,000	41 (9.7)	36 (8.5)	1	1

ETB, Ethiopian birr.

* Statistical significant.

### Factors associated with malaria prevention practices

No statistically significant variables were identified in the binary logistic regression; however, in the multivariable logistic regression, only one variable (poor knowledge) had a statistically significant association with poor malaria prevention practices. Study participants with poor knowledge were more likely to have poor malaria prevention practices than those with good knowledge (AOR = 1.85; 95% CI: 1.2–2.8; Table 8).

**Table 8:** Factors associated with malaria prevention practices.

**Variables**	**Categories**	**Prevention practices**		**COR**	**AOR**
		**Good**	**Poor**		
		***N*** **(%)**	***N*** **(%)**		
Household occupation	Pastoralists	61 (14.5)	254 (60.2)	0.5 (0.27–1.02)	0.56 (0.28–1.1)
	Agro pastoralist	12 (2.8)	95 (22.5)	1	1
Educational level	Illiterates	25 (5.9)	176 (41.7)	1.74 (0.921–3.3)	1.43 (0.74–2.8)
	Primary	27 (6.4)	88 (20.9)	0.81 (0.42–1.53)	0.76 (0.4–1.46)
	Secondary	21 (5)	85 (20.1)	1	1
Knowledge	Poor	19 (4.5)	132 (31.3)	1.73 (0.98–3.04)	1.85 (1.2–2.8)^*^
	Good	54 (12.8)	217 (51.4)	1	1
Attitude	Negative	34 (8.1)	190 (45)	1.37 (0.83–2.27)	1.16 (0.68– 1.96)
	Positive	39 (9.2)	159 (37.7)	1	1

* Statistical significant.

### Factors associated with healthcare-seeking practices for malaria prevention

In the binary logistic regression, participants with ages ranging from 18 to 25 years, poor knowledge, and a negative attitude were more likely to have poor healthcare seeking practices, but pastoralists were less likely to have poor healthcare seeking behavior. In the multivariable logistic regression analysis, participants with ages ranging from 18 to 25 years old (AOR = 3.5, 95% CI: 1.73–7.1) were nearly three times more likely to have poor healthcare seeking behavior than those with ages >45. Pastoralist participants (AOR = 0.46; 95% CI: 0.28–0.8) were 54% less likely to have poor healthcare seeking practices than agro-pastoral participants (Table 9).

**Table 9:** Factors associated with care-seeking practices to prevent malaria.

**Variables**	**Categories**	**Care seeking practices**		**COR**	**AOR**
		**Good**	**Poor**		
		**No (%)**	**No (%)**		
Age	18–25	29 (6.9)	56 (13.3)	3.46 (1.8–6.8)^*^	3.5 (1.73–7.1)^*^
	26–35	105 (24.9)	63 (14.9)	1.08 (0.6–1.94)	1.2 (0.63–2.2)
	36–45	63 (14.9)	39 (9.2)	1.12 (0.59–2.1)	1.3 (0.66–2.5)
	≥46	43 (10.2)	24 (5.7)	1	1
Marital status	Married	156 (37)	108 (25.6)	0.58 (0.25–1.36)	0.53 (0.21–1.3)
	Single	73 (17.3)	61 (14.5)	0.71 (0.3–1.69)	0.55 (0.2–1.36)
	Divorced	11 (2.6)	13 (3.1)	1	1
Occupation	Pastoralists	194 (46)	121 (28.7)	0.47 (0.3–0.73)^*^	0.46 (0.28–0.8)^*^
	Agro pastoral	46 (10.9)	61 (14.5)	1	1
Educational status	Illiterates	95 (22.5)	106 (25.1)	1.92 (1.18–3.1)	1.27 (0.75–2.15)
	Primary	78 (18.5)	37 (8.8)	0.82 (0.47–1.42)	0.71 (0.4–1.27)
	Secondary	67 (15.9)	39 (9.2)	1	1
Knowledge	Poor	74 (17.5)	77 (18.2)	1.65 (1.1–2.5)^*^	1.43 (0.92–2.2)
	Good	166 (39.3)	105 (24.9)	1	1
Attitude	Negative	116 (27.5)	108 (25.6)	1.56 (1.06–2.3)^*^	1.33 (0.86–2.01)
	Positive	124 (29.4)	74 (17.5)	1	1

* Statistical significant.

## Discussion

Understanding malaria transmission and preventative measures at the individual and household level, which depend on populations' knowledge of the disease (13), attitude toward the disease, and implementation of prevention activities (11), are essential strategies to stop the spread of malaria in malaria-endemic areas (14). There must be a reduction in the number of malaria cases and deaths at the global (1–5), regional (6, 7), national (8, 9), and district (9) levels. Therefore, this study assessed malaria prevention knowledge, attitude, and practice among households living in malaria-endemic areas.

In this study, 64.2% of household heads had good knowledge of malaria prevention. This magnitude is lower than the previous studies in sub-Saharan Africa (15), Nigeria (17), Cameroon (18), Mozambique (21), and Ethiopia (25). The discrepancy could be due to educational status, i.e., the presence of a high rate of illiteracy in the study region (38), and the COVID-19 pandemic may also have disrupted malaria prevention efforts by making it more difficult to provide health education about the symptoms, transmission, and prevention of malaria (5), and the study areas, which are rural pastoral areas. However, compared to earlier studies conducted in Senegal (16), Burkina Faso (19), South Africa (20), Tanzania (22), and southern Ethiopia (23), the magnitude of the current study's knowledge of malaria prevention was higher. This variation could be due to different study participants, such as in age group and gender, as well as different outcome measures or definitions and different study periods. Another reason could be that long-term residence in a malaria-endemic region exposed study participants to the disease's symptoms and transmissions there. The current findings on knowledge of malaria prevention were consistent with a study conducted in the Amhara region of Ethiopia (24).

In the current finding, 46.9% of study participants exhibited a positive attitude. This magnitude is lower than previous studies conducted in African nations (13, 15–18, 20). The reasons for this variation could be due to different study participant characteristics, such as the fact that some participants were adults in some studies (17, 20, 22), adolescents or children in others (15, 16), and only women in another study (18), as well as the pastoralist life styles and cultures of the current study participants. The other reasons may be variations in outcome measurement, the disruption of malaria prevention and control efforts as a result of COVID-19 (5), and differences in study setting. Furthermore, the current study's magnitude of attitude toward malaria prevention was lower than studies conducted in different parts of Ethiopia (23–25). The reason for this discrepancy may also be that the use of different tools to measure attitude and the effect of COVID-19 on health services led to the neglect of malaria prevention efforts (5).

The present study showed that 17.3% of participants had good malaria prevention practices (bed net use). This result is significantly lower than previous studies conducted in the Africa nations (15–19, 21, 22), as well as studies in Ethiopia (23–25). The difference may be the measurement of malaria prevention practices; in the current study, only bed nets were used to measure malaria prevention practices, but other malaria prevention activities may have been used in other studies. The adoption of traditional methods (27) and self-medication (26) may be other factors contributing to this disparity. Despite the fact that using bed nets to combat malaria is an efficient method in places where the disease is endemic (11), only a few households among the study participants were using bed nets, which may be a reason for the high malaria prevalence in the region (9). The other reason for these poor malaria prevention practices may also be the COVID-19 pandemic, which disrupted malaria preventive services (5), leading to a challenge in distributing bed nets to households. Lack of access to healthcare services brought on by a scarcity of healthcare facilities and medical professionals could also impede households from engaging in malaria prevention (33). On the other hand, the magnitude of bed net use practices among the current study participants was higher than in a previous study in South Africa (20). The perceived favorability of indoor residual spray (76%) and other malaria preventive methods over bed netting among South African study participants may be the cause of this variation.

In this study, 56.9% of study participants had good healthcare seeking behaviors, which is lower than the previous studies conducted in Senegal (16) and Nigeria (17). Moreover, the percentage of study participants with good healthcare seeking behaviors was lower than an earlier study in the Afar region that stated that most Afar people (71%) preferred to receive their medical care from modern health facilities (27). The participants' favorable attitudes regarding the use of self-medication (26) and traditional medication practices may be the cause of the difference. The low healthcare seeking practices in the present study led to a decrease in the use of medication for malaria prevention strategies implemented in malaria-endemic areas (11). Due to these reasons and the study area's favorable environmental conditions (due to little rainfall) (2, 10), malaria transmission might have increased there (9). Therefore, the uptake of healthcare services should increase, and in turn, the use of antimalarial drugs should improve. Eighteen percent of study participants in this study never visited health facilities when they encountered malaria symptoms, which is comparable with the previous study conducted in the Afar region of Ethiopia, in which a total of 19% of participants (3% used self-medication and 16% used traditional practices) did not use health care facilities (27). The absence of health facilities and financial constraints may also be contributing factors for the 18% of the study participants who never went to a health facility when they experienced malaria symptoms (33).

In the current study, low-income and illiterate household heads were more likely to have poor malaria prevention knowledge than secondary school and high-income participants, respectively. This evidence is in line with the previous findings (16, 18, 19, 22–24). Government programs that can improve economic and educational opportunities should be established to advance malaria prevention knowledge in the Afar region. Poor malaria prevention knowledge was significantly associated with poor malaria prevention practices (poor bed net use). This evidence is consistent with the previous findings that poor knowledge resulted in poor malaria management (29). Household heads should be given community health education about malaria to get adequate knowledge of malaria prevention that aids in combating malaria (29).

Participants who were married or illiterate had a statistically significant association with a negative attitude toward malaria prevention. This finding is in line with a previous study (16). The participants' attitudes that are more supportive of traditional medicine (27) and self-medication (26) may be the cause of this negative attitude toward malaria prevention. Household heads in the age range of 18–25 years old were more likely to have poor healthcare seeking behaviors than older age participants (≥46 years). The implementation of age-specific malaria healthcare-seeking intervention strategies is necessary, with a particular emphasis on young adults. Pastoralist participants were also less likely to have poor healthcare-seeking behaviors.

The study's limitation is its cross-sectional nature, which reflects data obtained at a certain time point and may alter in subsequent periods, which can result in some bias, including recall bias. A cause-and-effect relationship was also not examined. A longitudinal study design should thus be conducted to examine the causality of the proposed relationship. It used the head of the household as a stand-in for the knowledge, attitude, and practices regarding malaria prevention that all household members possess. It would have been preferable to adopt a sample strategy that included a wider range of adults from the study community, but due to a lack of financing, this was not achievable. As a result, the findings could not accurately reflect the viewpoints of the entire community. Due to the fact that some of the data included in the study was self-reported by the participants, the estimates may have been overestimated or underestimated. For instance, the participants may have overstated the social desirability of bed net use because it was self-reported. Furthermore, using closed-ended questions may limit the range and depth of responses, omit relevant contextual information that isn't covered by the options, introduce researcher bias or other questionnaire-related factors, and reduce respondents' engagement and interest. The questionnaire wasn't also developed to evaluate the quality of bed nets, such as bed net insecticide and the presence of holes. However, this study is the first in the region where malaria transmission is high, and it is used as an input to establish intervention strategies to improve malaria prevention knowledge and increase uptake of bed nets. Health promotion-based interventional study designs (pre-test and post-test) should be carried out in the future to improve malaria prevention knowledge and attitude. This will help increase the uptake of bed net usage.

## Conclusion

Although the majority of participants had good knowledge of malaria prevention, there are still a significant number of participants with poor knowledge of malaria prevention. Nearly half of participants had good healthcare-seeking behavior and a positive attitude toward malaria prevention. Only a small percentage of individuals used bed nets regularly to avoid malaria. Economic reform and community health education were required to address issues with low-income and illiterate household heads' poor knowledge and married and illiterate participants' negative attitudes. Young adults' poor healthcare-seeking behavior should also improve in order to promote malaria prevention.

## Data availability statement

The original contributions presented in the study are included in the article/supplementary material, further inquiries can be directed to the corresponding author.

## Ethics statement

Ethical approval was obtained from a Research and Ethics Review Committee of the Health Science College, Samara University, Ethiopia. All methods were performed in accordance with the relevant guidelines and regulations. Informed consent was obtained from all the study participants. No one was harmed as a result of participating in this study. The studies were conducted in accordance with the local legislation and institutional requirements. The participants provided their written informed consent to participate in this study.

## Author contributions

DA: Conceptualization, Data curation, Formal analysis, Funding acquisition, Investigation, Methodology, Project administration, Resources, Software, Supervision, Validation, Visualization, Writing—original draft, Writing—review and editing. TG: Conceptualization, Data curation, Formal analysis, Funding acquisition, Investigation, Methodology, Project administration, Resources, Software, Supervision, Validation, Visualization, Writing—original draft, Writing—review and editing.
